# Choice Rules and Accumulator Networks

**DOI:** 10.1037/dec0000038

**Published:** 2015-07-27

**Authors:** Sudeep Bhatia

**Affiliations:** 1 Behavioral Science Group, Warwick Business School, University of Warwick

**Keywords:** decision making, heuristics, sequential sampling, multi-attribute choice, leaky competitive accumulation

## Abstract

This article presents a preference accumulation model that can be used to implement a number of different multi-attribute heuristic choice rules, including the lexicographic rule, the majority of confirming dimensions (tallying) rule and the equal weights rule. The proposed model differs from existing accumulators in terms of attribute representation: Leakage and competition, typically applied only to preference accumulation, are also assumed to be involved in processing attribute values. This allows the model to perform a range of sophisticated attribute-wise comparisons, including comparisons that compute relative rank. The ability of a preference accumulation model composed of leaky competitive networks to mimic symbolic models of heuristic choice suggests that these 2 approaches are not incompatible, and that a unitary cognitive model of preferential choice, based on insights from both these approaches, may be feasible.

One of the primary approaches to studying the cognitive underpinnings of decision making involves heuristic choice rules. Heuristics are short cuts for solving problems. Within the domain of decision making, heuristics specify simple algorithms for accessing and manipulating attribute values. For example, instead of considering the values of all alternatives on a given attribute, many heuristic rules only utilize rank-based information, such as information regarding which alternative is the best on the attribute. Although the behavior generated by heuristics often departs from strict economic rationality, many researchers consider heuristics to be accurate descriptions of how humans actually make choices (Gigerenzer & Goldstein, 1996; Gigerenzer, Todd, & the ABC Research Group, 1999; Gilovich, Griffin, & Kahneman, 2002; Marewski & Schooler, 2011; Newell & Simon, 1972; Payne, Bettman, & Johnson, 1993; Simon, 1956; Tversky, 1972; Tversky & Kahneman, 1974).

An alternate approach to studying decision making involves the accumulation of preferences, often modeled using neural networks (Bhatia, 2013, 2014; Bogacz, Usher, Zhang, & McClelland, 2007; Busemeyer & Townsend, 1993; Krajbich, Armel & Rangel, 2010; Rangel & Hare, 2010; Roe, Busemeyer, & Townsend, 2001; Usher & McClelland, 2004). According to this approach, decision makers sample attribute values sequentially, and integrate these values in neuron-like units representing preferences for choice alternatives. Decisions are made when the preference for an alternative crosses a threshold level of activation. Many of these models also assume leaky competitive accumulation (Roe et al., 2001; Usher & McClelland, 2004; see also Usher & McClelland, 2001), that is lateral inhibition between preference nodes, and the feedback of information from these nodes to themselves—two biological properties that allow these networks to explain a wide range of human behavior.

These two important and influential approaches are usually studied separately. This is because of differences in the types of representations and computations that they assume are active in the decision process. Theories of heuristic choice are symbolic, consisting of sophisticated decision rules applied to abstracted variables, whereas preference accumulation is often best understood using dynamic, stochastic connectionist models, constrained by the simple structure of human neural circuitry. Both approaches warrant merit, and a convergence of these approaches is desirable. Such a convergence can contribute greatly to the goal of theory integration in decision making, and in doing so, shed light on the elementary information processing mechanisms that guide decision making across different domains. Beyond this, it can provide important organizing principles for the study of heuristics, and can subsequently constrain the range of possible heuristic rules at play in any given scenario. By specifying heuristic rules using a framework that is naturally suited to making probabilistic choice predictions and predictions regarding decision time, such a convergence can also greatly enhance the descriptive scope of the heuristics approach.

A convergence of heuristic and accumulator models can also enrich models of preference accumulation. Current accumulator networks, for example, are unable to explicitly model sophisticated attribute comparisons and transformations, such as those based on relative rank. Examining how these networks could be modified to incorporate this type of heuristic computation would contribute to the creation of more detailed and realistic models of preference accumulation.

We present a model of multi-attribute choice that allows for a convergence of heuristic and accumulator models. As with prior work on preference accumulation, we assume that preferences are represented in leaky competitive neurons that integrate attribute values over time. Unlike this work, we make explicit assumptions about the representation of the attributes that the available alternatives are composed off. Particularly, we assume that information about the values of available alternatives on various attributes is processed in different attribute sublayers, and that these attribute sublayers, like the preference accumulation layer, feature leakage and competition. Finally we assume that all nodes in the network have piecewise-linear activation functions bounded at 0 and 1.

Overall, our model is a two layered neural network with recurrent connectivity within each layer. This connectivity generates rich and complex dynamics, which for various connection weights can emulate a variety of well-known heuristic rules. Unlike many previous heuristic models, the behavior of proposed model is probabilistic, generating heuristic choice predictions that are vulnerable to error. This behavior is also dynamic, and the proposed model can easily be used to make decision time predictions. Our model is of course not only a model of heuristic choice: Specific parameter combinations in our model allow it to mimic existing accumulators as well.

## Heuristic Choice Rules

Consider a simple multi-attribute multi-alternative choice. A prosaic example of this problem involves the selection of a car. Each available car is defined on a number of attributes, such as mileage, horsepower, price, carbon emissions and so on. Rational decision making requires the evaluation of each attribute in each available car, the subsequent aggregation of each car’s attribute values, weighted by the importance of these attributes, and finally the selection of the car with the highest total value.

This problem can be very difficult. As the number of available alternatives or the number of attributes increases, more calculations are required, and identifying the highest value alternative becomes increasingly time consuming and effortful. It is in this setting that decision makers are likely to use heuristic rules, or cognitive shortcuts, to make their selections. The relevance of heuristic rules for preferential choice was first noted by Simon (1956), who proposed bounded rationality as a behavioral alternative to standard, value maximizing rationality. According to Simon, decision makers do not use all the information available to them in any given choice task; rather, they are likely to use only a subset of this information, manipulated in ways that are computationally feasible for humans. Heuristic rules, according to Simon, specify these manipulations, and are therefore the appropriate descriptors of the cognitive underpinnings of choice behavior (see also Simon, 1955; Newell & Simon, 1972).

The idea of heuristic rules as appropriate descriptors of choice is the foundational assumption in many other approaches as well. Tversky and Kahneman’s (1974) heuristics and biases framework, for example, proposes that heuristics form the basis of decision making, and that the use of these heuristics can lead to irrational behaviors (Gilovich et al., 2002; Tversky & Kahneman, 1974). The fast and frugal framework, proposed by Gigerenzer and colleagues (Gigerenzer & Goldstein, 1996; Gigerenzer et al., 1999), also assumes that heuristic rules underlie choice, though, unlike the heuristics and biases approach, the focus here is on formally modeling heuristics, and additionally examining the conditions where the use of heuristics is beneficial to decision making. An emphasis on the advantages of heuristic decision making is also a property of the adaptive decision maker framework, which examines the ways in which different heuristic rules can reduce the effort involved in preferential choice (Payne et al., 1993).

In this article, we attempt to implement some of the specific heuristic rules proposed in these (and other) frameworks in dynamic, stochastic connectionist networks. As there are a large number of qualitatively distinct heuristic rules that are currently studied, two restrictions to our task are in order. First, we limit ourselves to studying heuristic rules in preferential choice involving the manipulation and aggregation of attribute values, and will not study heuristics based on emotional or social cues. Second, we will consider only heuristics using attribute based processing, in which the values of several alternatives on a single attribute are processed before the examination of a second attribute. This is in contrast to alternative based processing, in which the values of several attributes in a single alternative are processed before the examination of a second alternative. Most heuristic rules for preferential choice rely on attribute based processing. Additionally, attribute based processing is considered to be cognitively simpler (Russo & Dosher, 1983), and has been shown to be efficient in settings involving uncorrelated attribute structures, and in settings requiring quick decisions (Payne, Bettman, & Johnson, 1988).

Given these two restrictions, we can now select a number of well-known heuristics to study. These are as follows:

1 Lexicographic. The lexicographic heuristic (LEX) is one of the earliest and best known choice rules. According to LEX, decision makers select the choice alternative with the highest value on the most important attribute (Fishburn, 1967; Gigerenzer & Goldstein, 1996; Hogarth & Karelaia, 2005a, 2007; Tversky, 1969).
2 CONF. The CONF heuristic is a variant of LEX. According to CONF, decision makers sample the attributes until an alternative that is the best on two attributes emerges. This alternative is then selected (Karelaia, 2006). The CONF heuristic can be extended to a more general choice rule, k-CONF, which requires the selected alternative to have the highest value on *k* attributes.
3 Weighted pros. According to weighted pros heuristic (WP), all attributes are sampled and each alternative is classified as best, with a value of one, or not best, with a value of zero, on each attribute. These binary values are then weighted and added, and the alternative with the highest total weighted sum is selected (Huber, 1979).
4 Majority of confirming dimensions. The majority of confirming dimensions (MCD) heuristic (also known as the tallying heuristic) is nearly identical to WP: it samples all attributes and processes only information pertaining to the best alternative on each attribute. It also, however, further simplifies the choice process by using equal weights for all attributes. According to MCD, the alternative that is the best on the most number of attributes is the one that is selected (Russo & Dosher, 1983).
5 Equal weights. The equal weights heuristic (EW) involves the sampling of all attributes and the consideration of the values of all alternatives on these attributes. Each attribute is, however, given an equal weight, so that the alternative with the highest total (nonweighted) attribute value is selected (Dawes, 1979; Dawes & Corrigan, 1974).
6 Weighted additive. The weighted additive rule (WAD) is not strictly a heuristic. Rather it is a rule that leads to the selection of the most desirable alternative (when the correct weights are known for certain). According to WAD decision makers sample, weigh and aggregate the attribute values of all alternatives.

## Three Principles of Bounded Rationality

There are three principles of bounded rationality that are particularly useful for characterizing heuristic decision making. All three of these principles reduce the amount of information processed in the decision task, and together they form the basis of a large number of choice rules involving attribute based processing, including the ones discussed above. The first principle involves the examination of fewer attributes and fewer alternatives: when attributes are examined sequentially, as with attribute based processing, decision makers often accept an alternative after only a few attributes have been sampled. As the order in which these attributes are sampled is often independent of the choice set, this principle also implies that interattribute dependencies at play in the choice task are ignored. The second principle involves the simplification of attribute weighting: instead of ascribing different weights to different attributes, decision makers often give all attributes the same weight in their decision. The third principle involves less complicated attribute comparisons across alternatives: instead of processing the exact values of all alternatives on a particular attribute, decision makers often identify only the best alternative on that attribute, and use this to accept alternatives in the choice set (see also Gigerenzer & Brighton, 2009; Shah & Oppenheimer, 2008).

These principles can all be observed in the heuristics listed in the previous section. The first principle, for example, is satisfied by LEX and CONF, which reduce the effort and time required to make a decision, by limiting the total number of attributes that are sampled, and by ordering sampling probabilities independently of interattribute dependencies. We can observe the second principle at play in MCD and EW. Whereas these heuristics sample all attributes, each attribute is weighted equally in the subsequent decision. The final principle is satisfied by LEX, CONF, WP, and MCD. Instead of processing the values of all alternatives on a particular attribute, these heuristics only identify and process information about the best alternative on the attribute. Ordinal comparisons reduce the amount of information needed to make a decision, thereby simplifying the choice and reducing effort, and potentially time. Note that WAD does not satisfy any of these principles. As it leads to the selection of the most desirable alternative from the available choice set, it needs to process all of the information available in the decision task.

Although the three principles mentioned in preceding text are useful in explaining the key components of various heuristic choice rules, none of these principles are fundamentally rule based or symbolic in nature. Information reduction by considering fewer attribute and alternatives, by giving each attribute an equal weight, or by making ordinal comparison within attributes, can be implemented by a variety of cognitive models. Indeed a model that can instantiate these three principles may also be able to generate the many heuristic choice rules that are based on these principles.

## Preference Accumulation Networks

Connectionist networks provide a powerful approach to studying the decision process (Bhatia, 2013, 2014; Bogacz et al., 2007; Busemeyer, Jessup, Johnson, & Townsend, 2006; Glöckner & Betsch, 2008a; Roe et al., 2001; Usher & McClelland, 2004). Many of these networks involve leaky competitive accumulation, with self-feedback within preference nodes and lateral inhibition between preference nodes. These networks also use decision thresholds to determine choice, with choice options being chosen if their corresponding accumulator node exceeds a certain level of activation.

There are many benefits to using accumulation networks to study preferential choice. Besides being neurally plausible, the preference accumulation framework can instantiate optimal speed–accuracy trade-offs, as a special case (Bogacz, Brown, Moehlis, Holmes, & Cohen, 2006). Beyond this, preference accumulation is closely related to sequential sampling approaches in other domains, such as perceptual choice (Link & Heath, 1975; Usher & McClelland, 2001), sensory detection (Smith, 1995), memory retrieval (Ratcliff, 1978), categorization (Nosofsky & Palmeri, 1997), and lexical choice (Ratcliff et al., 2004). Finally, theories of preference accumulation have an extensive descriptive scope. They have been used to explain behavioral findings as diverse as context dependence, reference dependence, risky choice, response time effects, speed–accuracy trade-offs, and task framing (Bhatia, 2013, 2014; Busemeyer & Townsend, 1993; Diederich, 1997, 2003; Johnson & Busemeyer, 2005; Krajbich et al., 2010; Roe et al., 2001; Tsetsos, Chater, & Usher, 2012; Usher & McClelland, 2004), and stochastic accumulation based computations have been observed in the brain (Basten, Biele, Heekeren, & Fiebach, 2010; Hare, Schultz, Camerer, O’Doherty, & Rangel, 2011; Lim, O’Doherty, & Rangel, 2011).

An influential theory of how preference accumulation can describe individual decision making has been proposed by Busemeyer and Townsend (1993), in a model titled decision field theory (DFT). Since its publication, DFT has been implemented in a connectionist network (Busemeyer et al., 2006; Roe et al., 2001), and a number of closely related theories have likewise formalized preference accumulation within connectionist networks (Bhatia, 2013; Bogacz et al., 2007; Usher & McClelland, 2004). All of these approaches can easily instantiate the first principle of bounded rationality discussed above: decision thresholds allow for the consideration of only a few attributes, greatly simplifying the decision process. Indeed Lee and Cummins (2004) have already shown how an accumulation model can capture lexicographic decision making in binary choice (though their implementation cannot be extended to the general case with more than two choice alternatives).

Whereas these theories provide valuable insights about how preferences are represented and computed, they do not generally study the representation and computation of the attribute values that are accumulated into preferences. It is unclear, for example, how existing accumulator networks could be modified to integrate attribute ranks rather than continuous attribute values. These details are required for a complete theory of preferential choice. In this article, we formalize the representation of different attributes in separate neural layers, each with leakage and competition (an assumption traditionally applied only to preference representation). We show that formalizing attribute representation in this manner can instantiate the second and third principle of bounded rationality, thereby generating behavior that mimics that specified by the heuristic rules discussed in this article.

## Model

Let us consider choices between a set of alternatives defined on *M* attributes. The value of alternative *i* on attribute *j* is written as *x*_*ij*_, and weight given to attribute *j* is written as *w*_*j*_. Given this, the weighted total value of alternative *i* is $U_{i}=\sum_{j=1}^{M}w_{j}\;\cdot \;x_{ij}$ . The most desirable alternative in a choice set is the one with the largest value of *U*_*i*_. This is the alternative that would be selected by decision makers were they completely rational, and is the alternative predicted to be chosen by the WAD rule. Without loss of generality, we will assume that 0 < *x*_*ij*_ < 1 for all *i* and *j*.

The decision process is implemented in a two layer neural network. The first layer formalizes attribute representation, and consists of *M* sublayers, with each sublayer corresponding to each of the *M* different attributes that the alternatives are defined on. We assume that each sublayer itself consists of *N* nodes, with each node representing the value of a possible choice alternative on that attribute. The values of the alternatives available in a particular task, on an attribute, are represented by a subset of nodes in that attribute’s corresponding sublayer. The second layer represents preferences, and consists of *N* nodes, each representing the preference for a possible choice alternative. Preferences for the alternatives available in the task at hand are represented by a subset of nodes within this layer.

We assume that every node in this network has the same piecewise linear activation function, bounded at 0 and 1. More specifically, given an input *y*, the activation of a node is determined by the function *f*(*y*), with

$$f\left(y\right)=\left\{ \begin{array}{cc} 1 & \;\;\;\;\;\;\;y>1\\ y & 0\leq y\leq 1\\ 0 & \;\;\;\;\;\;\;y<0 \end{array}\right.$$ 1

We assume that the nodes in the attribute layer corresponding to the considered alternatives receive exogenous inputs based on the amounts of these attributes in the available alternatives. Thus the node representing the value of attribute *j* in alternative *i* in the first layer receives inputs *x*_*ij*_ if *i* is available in the choice set. If an alternative is not a part of the available choice set then the inputs to its attribute nodes are zero. We also assume that connections between the sublayers in the first layer and the second layer are equal to the weight given to the attribute represented in the sublayer. Particularly, the connection from the node representing attribute *j* in alternative *i*, to the node representing the preference for alternative *i*, is *w*_*j*_. 

In addition to connectivity between the layers, we assume connectivity within the layers. This connectivity involves leakage and competition. Particularly, as with prior preference accumulation networks, nodes in the preference accumulation layer are assumed to excite themselves, with self-feedback parameter *s*_*P*_, which is identical across all preference nodes. Nodes in this layer are also assumed to inhibit other nodes, with inhibition parameter *l*_*P*_, also identical across all preference nodes. Unlike prior networks, however, we assume that this type of connectivity is also active within each attribute representation sublayer with, *s*_*Aj*_ and *l*_*Aj*_ representing self-feedback and inhibition in the attribute representation sublayer *j*. If we write the activation of node *i* in the preference accumulation layer, or any given attribute representation sublayer, at time *t* (in the time scale of the layer) as *X*_*i*_(*t*), the inputs to node *i* from other layers, at time *t*, as *Y*_*i*_(*t*), and the self-feedback and lateral inhibition parameters of that layer as *s* and *l*, then we have:

$$X_{i}(t)=f[s\cdot X_{i}\left(t-1\right)-l\cdot \sum_{k\neq i}X_{k}(t-1)+Y_{i}\left(t\right)]$$ 2

We shall assume that the attribute representation layer operates at a different time scale relative to the other layers, with the attribute representation layer reaching its equilibrium activation almost immediately. This allows us to use the stable equilibrium activation states of the nodes in each attribute sublayer, as that sublayer’s outputs, greatly simplifying our understanding of the network’s properties.^1^ This assumption is not just mathematically convenient, it is also justified by the functional differences between these layers: the preference accumulation layer holds information pertaining to the considered alternatives in memory so that these alternatives can be compared to make a decision, whereas the attribute representation sublayers retrieve information pertaining to stored attribute values. It is not unlikely that these layers differ in terms of their structural properties, such as the time they take to reach their equilibrium activation state.

Prior preference accumulation theories have assumed that each attribute’s values are sampled and accumulated stochastically and sequentially. We shall maintain this assumption: at each time step (in the time scale of the preference accumulation layer), one attribute sublayer is selected at random, and its stable activation states serve as weighted inputs to the preference accumulation layer. This process repeats at the next time step. In this article, we will refer to this time scale as the time scale of attribute sampling. We will also assume that attribute sampling is uniform and independent, with each attribute equally likely to be sampled. This can be easily modified to incorporate insights from related approaches that assume that attribute sampling is a function of the available choice set (e.g., Bhatia, 2013 see also Bhatia, 2014).

**Figure 1 fig1:**
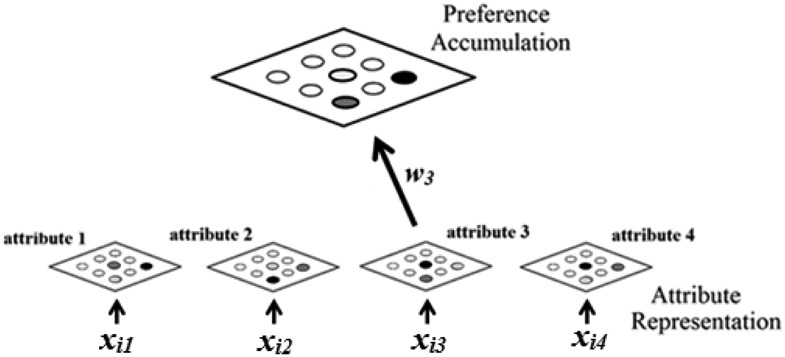
The proposed network, which consists of two layers corresponding to attribute representation and preference accumulation, with the attribute representation layer further divided into *M* sublayers, each corresponding to an attribute. The preference accumulation layer and the attribute representation sublayers each consist of *N* nodes, with each node corresponding to a possible choice alternative. Additionally, each layer has self-feedback and lateral inhibition, and attribute sublayers are sampled sequentially (with the third sublayer beings sampled here).

Finally, we need to specify the decision rules used by this network. Prior work has assumed that alternatives are chosen when the activation of their corresponding preference node goes above a fixed acceptance threshold. Again, we will maintain this assumption, and will write the acceptance threshold as *Q*. The first alternative to cross *Q* is the one that is selected. Figure 1 illustrates the structure of the proposed network. [Fig-anchor fig1]

Let us now examine a decision. Before the decision begins, all nodes in the network are off, and all inputs to these nodes are 0. At the start of the decision, the nodes in the attribute sublayers corresponding to the available alternatives receive inputs *x*_*ij*_. These inputs are passed through the activation function *f* to determine the activation in the different layers. As the time scale on the attribute sublayers is much quicker than the time scale of attribute sampling, the attribute sublayers stabilize immediately. The stable activation state for nodes corresponding to nonavailable alternatives is 0, as these nodes do not receive any exogenous inputs. The stable activation states for nodes corresponding to available alternatives in layer *j* depend on *s*_*Aj*_ and *l*_*Aj*_, as well as the values of all the other alternatives on attribute *j*.

Some attribute is now sampled at random, and the preference accumulation nodes corresponding to available alternatives receive inputs equal to the weighted activation states of their corresponding nodes this attribute’s sublayer. These are passed through the activation function *f* to generate the preference activations for the alternatives at *t* = 1. Note that the preference nodes corresponding to nonavailable alternatives will get no inputs and thus will remain off. If the activation of a preference node crosses the acceptance threshold, *Q*, then its corresponding alternative is selected and the decision terminates. If not, then this process repeats from the beginning. Particularly, another attribute is sampled, and the weighted equilibrium activations of the nodes in this attribute’s sublayer enter as inputs into corresponding preference accumulation nodes. Preference accumulation nodes also receive excitatory inputs from themselves from the previous time period, weighted by *s*_*P*_, and inhibitory inputs from competing preference nodes, weighted by *l*_*P*_. Acceptance decisions are made based on whether the resulting activation values of these preference nodes cross the threshold, and this process repeats itself until the decision terminates.

## Properties

The type of processing that the proposed model accomplishes is similar to that of a standard preference accumulation network. Particularly, this network stochastically samples, weighs and accumulates information regarding attribute values into preferences. Recurrent connectivity and threshold decision making in the preference accumulation layer generate rich dynamics that can be used to model the time course of the decision process. Additionally, connections between the preference accumulation and attribute representation layer weigh the outputs of the attribute representation layer proportionally to attribute importance. Finally, recurrent connectivity in the attribute representation layer serves to modify the attribute values that are used in the accumulation process. Although this connectivity is based on the same principles as that in the preference accumulation layer, we assume that it operates on a fast enough time scale that we do not have to worry about the time dynamics of attribute representation. The modified attribute values that are eventually accumulated, are simply the equilibrium activation states generated by processing actual attribute values in the attribute sublayers.

We can understand the emergent properties of this network if we understand the types of computations performed by these components of the network. The followings sections will explore how these computations depend on the connection weights between and within the layers, and will demonstrate how varying these connections can lead to computations specified by the three principles of bounded rationality, subsequently generating behavior predicted by each of the specific heuristic rules explored in the article. These sections will also discuss the relationship between the parameters of the model and randomness in its behavior, as well as the effect of this randomness on heuristic behavior.

### The Preference Accumulation Layer

Recurrent connectivity and threshold decision making in the preference accumulation layer produce computations resembling the first principle of heuristic choice. Appropriate values of *Q* lead to the acceptance of alternatives before all attributes have been sampled, and self-feedback and lateral inhibition, in conjunction with the specific thresholds in use, perform the computations guiding this acceptance. This reduces the number of attributes and alternatives considered during the decision, subsequently reducing the effort, time and complexity involved in the decision.

Self-feedback for example, determines the dependence of the decision at a particular time period on information sampled in previous time periods. If *s*_*P*_ = 0, then weighted information regarding attribute values for each alternative, sampled in any particular time period, is considered independently from similar information sampled in previous time periods. This is a property of heuristics such as LEX, which specifies the acceptance of an alternative based only on absolute or relative attribute values of the attribute sampled in that time period. In contrast, if the self-feedback term *s*_*P*_ = 1, then weighted information regarding attribute values for each alternative, sampled in any particular time period, is added to similar information sampled in previous time periods. This is a property of heuristics such as CONF, WP, MCD, and WAD, which specify the acceptance of an alternative based on all sampled the attribute values.

Thresholds in the preference accumulation layer operate in conjunction with the self-feedback parameters, as well as the outputs of the attribute representation layer, to instantiate specific acceptance rules. If, for example, the attribute representation layer can identify the best alternative on an attribute (as, e.g., required by LEX) by having its equilibrium activation in that attribute’s sublayer equal to 1, and the equilibrium activation of all other alternatives less than 1, then if we set *s*_*P*_ = *l*_*P*_ = 0, and choose an acceptance threshold *Q* = max(*w*_*j*_), our model would be able to select the best alternative on the most important attribute.

Different thresholds do not only allow us to instantiate the various properties of heuristic rules, they also allow us to control the time that it takes to make a decision, and subsequently modify the level of noise that characterizes each decision. As with other models of preference accumulation, stochastic attribute sampling generates variability in the change in preference, and the effect of this variability is greater if preferences are allowed to accumulate for only a short period of time. Decisions are extremely noisy (and take very little time) if the decision thresholds are relatively small compared with the inputs to the preference accumulation layer.

Lateral inhibition can be seen to have a similar role in the model. Whereas self-feedback establishes a dependency between the preference activation for an alternative in one time period and its activation in the previous time period, lateral inhibition establishes a dependency between the preference activation for an alternative in one time period and the activation of other alternatives in the previous time period. As lateral inhibition involves negative connection strengths, the preference for an alternative reduces with higher preferences for its competitors. This allows the decision maker to accumulate information pertaining to relative preferences (rather than absolute preferences) for the competing alternatives. Relative accumulation makes it more likely that the accumulator node that receives the highest input is the one that is chosen. Whereas many existing accumulator models feature lateral inhibition between their accumulators, we will, for simplicity, avoid this assumption in our analysis. This is because the key insights of this article do not depend critically on lateral inhibition in the preference accumulation layer.

Finally note that the above dynamics also ignore interattribute dependencies. As attribute sampling is independent of the choice set, and because the preference accumulation layer aggregates information about different attribute identically (i.e., with the same decay and inhibition), the model does not process the desirability of an attribute conditional on the other attributes in the choice alternative.

### Attribute-Preference Connection Weights

The connections, *w*_*j*_, between the attribute representation and preference accumulation layers serve perhaps the simplest role in the proposed network. These connections merely reweigh the equilibrium activations of the attribute sublayer before these activations are aggregated by the preference accumulation nodes. If all connections are equal, then each attribute would be given an equal weight, as, for example required by MCD or EW. If connections correspond to attribute importance, then different attributes will receive different weights, as required by WP or WAD. This corresponds to the second principle of heuristic choice.

These connections also play a role in heuristics that do not sample all attributes. Here, however, they do not necessarily facilitate the weighted aggregation of attribute values, rather they modify the rules that are applied to each attribute. Connection weights are identical for all attribute sublayers, for rules such as CONF, where the acceptance criteria do not vary based on the attribute that is sampled. Connection weights vary for different attributes sublayers, for heuristics such as LEX, which specify the selection of an alternative only if it is the best on the most important attribute.

Connection weights also play another role in our model: They allow us to scale the inputs into the preference accumulation layer, controlling the extent of accumulation that is possible before node activation in this layer hits its upper or lower bounds. Very small connection weights allow the network continue accumulating evidence for longer periods of time. This, as discussed above, reduces the amounts noise in the decision while also increasing the amount of time required by the model to make a decision.

### Attribute Representation Sublayers

The leaky competitive connectivity in the attribute representation sublayers is able to instantiate the third principle of heuristic choice. Varying values of self-feedback and inhibition in these layers can transform inputs pertaining to absolute attribute values in the alternatives, *x*_*ij*_, into activation states identifying the best alternatives on these attribute values. These types of ordinal comparisons on attributes underlie heuristics such as LEX, CONF, WP, and MCD, and are a key component of the choice rules known to be at play in decision making.

To understand the properties of the attribute representation sublayers, one must recall that we are assuming that the processing dynamics on these sublayers are fast enough, so that only equilibrium activation states are accumulated when the appropriate attributes are sampled. This assumption allows us to conveniently characterize computations on each attribute sublayer in terms of a mapping transforming attribute value inputs into stable modified outputs, with the same dimensionality as the inputs. More formally the sublayer corresponding to attribute *j* receives an *N* dimensional input ***y***_***j***_ = (*y*_*1j*_, *y*_*2j*_, . . . , *y*_*Nj*_). Here we have *y*_*ij*_ = *x*_*ij*_ if alternative *i* is considered, and *y*_*ij*_ = 0 otherwise. Given the self-feedback and inhibition parameters *s*_*Aj*_ and *l*_*Aj*_, this input is transformed by the function α(***y***_***j***_, *s*_*Aj*_, *l*_*Aj*_), giving an *N* dimensional output α_***j***_ = (α_*1j*_, α_*2j*_, . . . , α_*Nj*_), corresponding to the stable activation states of the nodes in the sublayer. Both *y*_*ij*_ and α_*ij*_ are in the interval [0, 1] for all *i* and *j*, as we have 0 < *x*_*ij*_ < 1 and the activation function *f* is bounded at 0 and 1.

For convenience let us order our *n* considered alternatives in terms of decreasing values on attribute *j*. Hence ***y***_***j***_ can be written as ***y***_***j***_ = (*x*_*1j*_, *x*_*2j*_, . . . , *x*_*nj*_, 0, 0 . . . , 0), with 1 > *x*_*1j*_ > *x*_*2j*_ > . . . > *x*_*nj*_ > 0. Now, how do different values of *s*_*Aj*_ and *l*_*Aj*_ affect α and determine α_***j***_? Let us start our analysis with the simplest case, holding *s*_*Aj*_ = *l*_*Aj*_ = 0. As there is no self-feedback or inhibition, and the activation function is linear with slope equal to 1, in the interval [0, 1], the sublayer’s stable activation state will be equal to its inputs, and α will simply be the identity function α(***y***_***j***_, *s*_*Aj*_, *l*_*Aj*_) = ***y***_***j***_.

Let us now incrementally increase *s*_*Aj*_, and hold *l*_*Aj*_ constant at *l*_*Aj*_ = 0. As positive activation, weighted by *s*_*Aj*,_ feeds back from a node to itself, the equilibrium activation states of all nodes corresponding to considered alternatives increase. If *s*_*Aj*_ is very small, then the stable activation value of these nodes will be increased, but will still remain below the activation upper bound, 1. Now further increasing the value of *s*_*Aj*_ will increase α_*ij*_ for considered alternatives, ultimately causing the activation state of alternative *i* = 1 (the considered alternative with the highest *x*_*ij*_) to saturate at 1. At this point increasing *s*_*Aj*_ has no further effect on α_*1j*_. Increasing *s*_*Aj*_ does however alter α_*ij*_ for *i* > 1, ultimately causing the activation state of alternative *i* = 2 (the considered alternative with the second highest *x*_*ij*_) to also saturate at 1. Further increases to *s*_*Aj*_ will have the same effect on the remaining nodes: these nodes will saturate at 1, in order of the magnitude of their inputs. Eventually for large enough *s*_*Aj*_ we will have α_*ij*_ = 1 for all *i ≤ n*, and α_*ij*_ = 0 for all *i* > *n*; that is, nodes corresponding to considered alternatives will have the maximal activation, whereas nodes corresponding to nonconsidered alternatives will be deactivated.

What does the inhibition parameter do? To explore this, let us incrementally increase *l*_*Aj*_, and hold *s*_*Aj*_ constant at *s*_*Aj*_ = 0. A positive value of *l*_*Aj*_ leads to negative inputs from all positively activated nodes into all other nodes. This reduces the activation value of all positively activated nodes in the attribute sublayer. If *l*_*Aj*_ is very small, then the stable activation values for nodes corresponding to considered alternatives will be decreased, but not decreased strongly enough for any of these nodes to hit the activation lower bound, 0. Further increasing *l*_*Aj*_ will however further decrease α_*ij*_ for all of these alternatives, ultimately restricting the stable activation state of alternative *i* = *n* (the considered alternative with the lowest *x*_*ij*_) to 0. At 0 α_*nj*_ is stable, and does not send inhibitory inputs into other nodes. In this setting, the dynamics of the remaining attribute nodes resemble the dynamics generated if alternative *n* was not being considered. Even further increases to *l*_*Aj*_ will restrict the activation of the node corresponding to alternative *i* = *n* −1 to 0 and the dynamics of the layer will resemble the setting where both alternative *n* and *n*-1 are not considered. Eventually large enough values of *l*_*Aj*_ will keep all nodes deactivated, except for the node corresponding to alternative *i* = 1 (the node with the highest input).

The effects of self-feedback and inhibition on the equilibrium states in an attribute representation sublayer can thus be understood as follows. Increasing *s*_*Aj*_ increases the equilibrium activation of all nodes corresponding to considered alternatives. Eventually high enough values of *s*_*Aj*_ cause these nodes to saturate at the maximal activation, 1. This happens in order of the strength of their inputs, so that the node with the highest input saturates first (needing the lowest value of *s*_*Aj*_ to saturate), the node with the second highest input saturates second (needing the second lowest value of *s*_*Aj*_ to saturate), and so on. Inhibition has a similar effect. Increasing *l*_*Aj*_ decreases the equilibrium activation of all nodes corresponding to considered alternatives. Eventually high enough values of *l*_*Aj*_ cause these nodes to saturate at the lowest activation, 0. This happens in order of the strength of their inputs, so that the node with the lowest input saturates first (needing the lowest value of *l*_*Aj*_ to saturate), the node with the second lowest input saturates second (needing the second lowest value of *l*_*Aj*_ to saturate), and so on. Although we have explored the effects of *s*_*Aj*_ and *l*_*Aj*_ keeping one or the other fixed at 0, this insight also holds if both are varied together.

**Figure 2 fig2:**
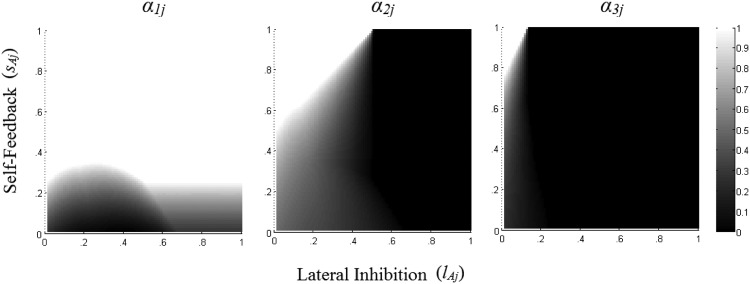
Equilibrium activation states for nodes in attribute representation sublayer *j*, as a function of *s*_*Aj*_ and *l*_*Aj*_. Here we have *x*_*1j*_ = 0.75, *x*_*2j*_ = 0.5, and *x*_*3j*_ = 0.25.

An illustration of this property is provided in Figure 2. Figure 2 considers three alternatives on an attribute *j*, such that *x*_*1j*_ = 0.75, *x*_*2j*_ = 0.5 and *x*_*3j*_ = 0.25. It varies *s*_*Aj*_ and *l*_*Aj*_ from 0 to 1, and displays the resulting equilibrium activation states of the nodes corresponding to these alternatives. These states are indicated by the shades on each of the three plots, with lighter shades corresponding to higher equilibrium activation states. Notice that increasing *s*_*Aj*_ always increases α_*ij*_ for all alternatives, whereas increasing *l*_*Aj*_ always decreases α_*ij*_ for alternatives 2 and 3, and almost always decreases α_*ij*_ for alternative 1. Alternative 1 is also never actually deactivated, unlike alternatives 2 and 3, which are deactivated for large enough values of *l*_*Aj*_. [Fig-anchor fig2]

**Figure 3 fig3:**
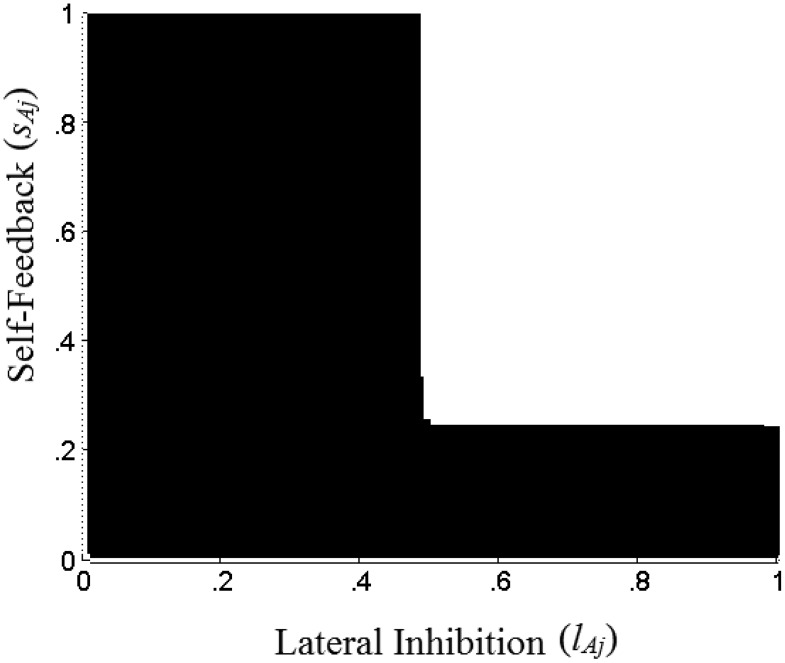
Parameter values identifying the best alternative on an attribute *j* (α_*1j*_ = 1, α_*2j*_ = 0 and α_*3j*_ = 0) in the attribute representation sublayer *j*, as a function of *s*_*Aj*_ and *l*_*Aj*_. White values indicate parameter values that can make the correct identification. Here we have *x*_*1j*_ = 0.75, *x*_*2j*_ = 0.5, and *x*_*3j*_ = 0.25.

We can use these insights to understand how the attribute representation layer can implement computations corresponding to ordinal comparisons on an attribute. Note that these computations require the attribute representation layer to set both α_*ij*_ = 1 for the best alternative and α_*ij*_ = 0 for other alternatives. Using the above insights we can show that high enough values of *s*_*Aj*_ and *l*_*Aj*_ ensure both that the best alternative saturates at 1, and that all other alternatives are suppressed to 0. Figure 3 demonstrates these insights, with the same choice options that are used in Figure 2. White areas on Figure 3 correspond to parameter combinations for which the network sublayer is able to compute the best alternative on attribute *j*, with equilibrium activations α_*1j*_ = 1, α_*2j*_ = 0 and α_*3j*_ = 0. Parameter combinations shaded black, give us either α_*2j*_ > 0 or α_*1j*_ < 1, or both. A formal demonstration of how, given any vector of inputs ***y***_***j***_, we can find some values of *s*_*Aj*_ and *l*_*Aj*_ that implement these computations, is provided in the Appendix (see the online supplemental Appendix).[Fig-anchor fig3]

### Implementing Heuristic Rules

Thus far we have shown how varying connection weights between and within the two layers of the proposed network allow the network to implement each of the three proposed principles of bounded rationality. Appropriate threshold, self-feedback and inhibition parameters, in the preference accumulation layer, permit the acceptance of alternatives before all attributes have been sampled. This leads to the examination of fewer attributes and alternatives than required by rational decision making, and corresponds to the first principle of heuristic choice. Connection weights between the attribute representation and preference accumulation layers specify attribute weighting, and can be used to implement attribute specific decision rules. Equal connection weights lead to equal attribute weighting (and the same decision rule for each attribute), corresponding to the second principle of heuristic choice. Finally the recurrent connectivity on the attribute representation sublayers allows the network to make ordinal comparisons of the alternatives on the various attributes. This simplifies the attribute values that are used in the decision process, corresponding to the third principle of heuristic choice.

Now each of the heuristic rules introduced in this article involves a specific combination of these three principles. Here we shall show that appropriate parameters in the proposed model can generate each of these specific combinations, leading to the accurate implementation of all of these rules. Note that we will explicitly derive the preference accumulation layer parameters (*s*_*P*_, *l*_*P*_, *Q*) and connection weights (*w*_*j*_) that are required to implement each of these rules. We will not however explicitly derive the required attribute representation layer parameters (*s*_*Aj*_, *l*_*Aj*_), as these parameters depend on the set of alternatives offered to the decision maker. Instead we will rely on the result introduced in the previous section, and shown in the Appendix (see the online supplemental Appendix), that states that for any set of considered alternatives there exist some values of *s*_*Aj*_ and *l*_*Aj*_ that allow attribute sublayer *j* to identify the best alternative in the set. Also note that the proposed model’s behavior is stochastic, whereas most of the heuristic rules considered are deterministic. Our implementation will demonstrate how the proposed model can be used to place these deterministic heuristic rules within a probabilistic framework.

#### Lexicographic

According to LEX, decision makers select the choice alternative with the highest value on the highest weighted attribute. Recall that for high values *s*_*Aj*_ and *l*_*Aj*,_ the attribute representation layer can identify the best alternative on an attribute. The outputs given by this layer, for attribute *j*, for these values of *s*_*Aj*_ and *l*_*Aj*_, are α_*ij*_ = 1 if alternative *i* has the largest amount of attribute *j*, and α_*ij*_ = 0 otherwise. If we set *s*_*Aj*_ and *l*_*Aj*_ for the attribute with the highest weight to be such that its layer is able to identify the best alternative, and additionally select an acceptance threshold *Q* = max(*w*_*j*_) and self-feedback parameter *s*_*P*_ = 0 then the alternative specified by LEX will be the one that is chosen by the network. This is because *s*_*P*_ = 0 will ensure that the network does not aggregate attribute values over time, but instead considers each attribute individually, independent of attributes sampled in the past. *Q* = max(*w*_*j*_) and our chosen values of *s*_*Aj*_ and *l*_*Aj*_ further ensure that a threshold is crossed only if the attribute that has the highest weight is sampled.

Note that lower values of *Q* than *Q* = max(*w*_*j*_) introduce noise into our decision. For example, if *Q* is equal to the second highest weight, then the object that is the strongest on the second most important dimension is equally likely to be chosen than the object that is the strongest on the most important dimension. Further reducing *Q* makes it more likely that alternatives that are the strongest on less important attributes are chosen, and very low values of *Q* lead to the selection of the alternative that is the best on the first sampled attribute.

Finally, note that as both *w*_*j*_ and attribute sampling probabilities are independent of the choice set, the proposed instantiation of the LEX heuristic cannot process interattribute dependencies: The specific attribute that plays the key role in this heuristic does not vary with the other attributes involved in the choice task. This is also a property of existing lexicographic heuristics, which involve the use of nonconditional cue validities as weights.

#### CONF

The CONF heuristic is a variant of LEX, and can be implemented using similar principles as LEX. According to CONF, decision makers sample the attributes until an alternative that is the best on two attributes emerges. This alternative is then selected. Recall that for high values of *s*_*Aj*_ and *l*_*Aj*,_ the computations on the attribute representation sublayer *j* can identify the best alternative on attribute *j* by giving outputs α_*ij*_ = 1 if alternative *i* has the largest amount of attribute *j*, and α_*ij*_ = 0 otherwise. Also recall that *s*_*P*_ = 1 and *l*_*P*_ = 0 allow for the addition of preferences over time, in the preference accumulation layer. If we set *w*_*j*_ = *w* < 0.5, and *Q* = 2 · *w*, then each alternative would increase by *w* if it is the best alternative on the sampled attribute, and increase by 0 if it is not. Because *s*_*P*_ = 1, preference accumulation would have perfect memory, and every increase will be added to every previous increase. Eventually, the alternative that is the first to be best on two attributes will have an activation 2 · *w* and will cross *Q*. This will lead to it being selected.

The generalization of CONF, k-CONF can also be implemented using this method. We would require the same values of *s*_*Aj*_ and *l*_*Aj*_ as in CONF, and furthermore set *w*_*j*_ = *w* < 1/*k*, and *Q* = *k* · *w*. With these parameters, the alternative that is the first to be the best on *k* attributes, will reach an activation level of *k* · *w*, crossing the threshold *Q*. This alternative will be the one that is chosen.

#### Weighted pros

WP requires the sampling of all attributes, and the classification of each alternative as the best, with a value of 1, or not the best, with a value of 0, on each attribute. These binary values are then weighted and added, and the alternative with the highest total weighted value is selected. This heuristic is quite easy to implement in our network. Unlike the heuristics discussed thus far, it samples all attributes and does not require the early acceptance of any alternative. We thus need to set the acceptance threshold *Q* to be sufficiently close to 1, and set our weights *w*_*j*_ to be sufficiently small (but proportional to the weights we wish to place on the attributes), so that a large number of attributes can be (repeatedly) sampled and accumulated before the decision is made. WP also requires interattribute comparisons that identify the best alternative on an attribute. Hence we need to choose high values of *s*_*Aj*_ and *l*_*Aj*,_ so that the attribute representation sublayer *j* gives outputs α_*ij*_ = 1 if alternative *i* has the largest amount of attribute *j*, and α_*ij*_ = 0 otherwise. Finally as we need to aggregate weighted information for all attributes, we can set *s*_*P*_ = 1. WP can now be implemented. Particularly, if attribute *j* is sampled at time *t*, the node corresponding to the considered alternative *i*, gets an input α_*ij*_ = 1 if alternative *i* is the best on attribute *j*, and an input of α_*ij*_ = 0 otherwise. This is weighted and added to the sum of similar inputs from previous time periods. Over time, the choice option specified by WP gets the highest activation and is most likely to cross *Q*.

Note again that *Q* determines that amount of noise in the network’s behavior. High values of *Q* with relatively small *w*_*j*_ generate nearly deterministic decisions, accurately implementing WP. In contrast, moderate values of *Q* generate probabilistic decisions, in which the modal choice is nonetheless the choice specified by WP. Finally small values of *Q* lead to choices that deviate significantly from those specified by WP.

#### Majority of confirming dimensions

MCD is nearly identical to WP: it samples all attributes and processes only information pertaining to the best alternative on each attribute. It also however further simplifies the choice process by using equal weights for all attributes. As a result, MCD can be implemented in a manner identical to WP, with the added assumption that attribute weights are equal, with *w*_*j*_ = *w* for all *j*. Additionally, as with WP, high values of *Q* generate deterministic behavior, moderate values of *Q* generate probabilistic behavior in which the choice specified by MCD is the modal response, and low values of *Q* generate probabilistic behavior that departs significantly from that predicted by MCD. Indeed low enough value of *Q* ultimately lead to the types of choices predicted by the CONF heuristic.

#### Equal weights

EW involves the selection of the alternative with the highest total nonweighted attribute value. EW can be implemented in much the same way as MCD, however as EW does not involve interattribute comparisons, we set *s*_*Aj*_ = *l*_*Aj*_ = 0. Thus at time *t* if attribute *j* is sampled, the attribute representation layer sends an output of α_*ij*_ = *x*_*ij*_ for the considered alternative *i*. This is weighted by the constant weighting term *w*_*j*_ = *w* and added to similar outputs summed over previous time periods in the preference accumulation node corresponding to alternative *i*. Once again, as with WP and MCD we need to set *Q* to be high enough to ensure that decisions are made with relatively little noise.

#### Weighted additive rule

According to WAD decision makers sample, weigh and aggregate the attribute values of all alternatives. As this rule does not require interattribute comparisons, it can be implemented by setting *s*_*Aj*_ = *l*_*Aj*_ = 0, to give outputs α_*ij*_ = *x*_*ij*_ from the attribute representation sublayer corresponding to attribute *j*, for the considered alternative *i*. Additionally *w*_*j*_ are set proportional to the weights we wish to place on required attributes. Finally, we set *s*_*P*_ = 1 so that all attribute values are aggregated over time, and a high value of *Q* so that the decision is relatively noiseless.

### Additional Attribute Transformations

One of the key insights that drive the results in this article pertain to ordinal comparisons on the attribute representation sublayers: We have shown that high self-feedback and high lateral inhibition within these layers allow them to identify the strongest alternative on an attribute by having its stable activation at 1, and the stable activation of all other alternatives at 0. In contrast, when there is no feedback or inhibition within these layers, they are able to process the attribute values of all alternatives without transforming them based on their rank.

Leakage and competition on these sublayers are, however, able to accomplish more than just an identify-the-best transformation. These layers can, for example, also identify the worst alternative on an attribute by having its stable activation at 0, and the stable activation of all other alternatives greater than 0. This type of comparison is a property of heuristics such as the elimination by least attractive heuristic, which samples attributes sequentially and eliminates the alternative that is the lowest valued on the sampled attribute (Svenson, 1979).

To see how the proposed model can perform these types of transformations recall again that increasing *l*_*Aj*_ decreases the equilibrium activation of all nodes corresponding to considered attribute, with high enough values of *l*_*Aj*_ causing these nodes to saturate at the lowest activation, 0. This happens in order of the strength of their inputs, so that the node with the lowest input saturates first, needing the lowest value of *l*_*Aj*_ to saturate. If we choose moderate values of *l*_*Aj*_, values that are high enough to suppress the node corresponding to alternative *n* to 0, but not high enough to suppress a node corresponding to alternative *i* < *n* to 0, then we can easily capture this type of processing.

**Figure 4 fig4:**
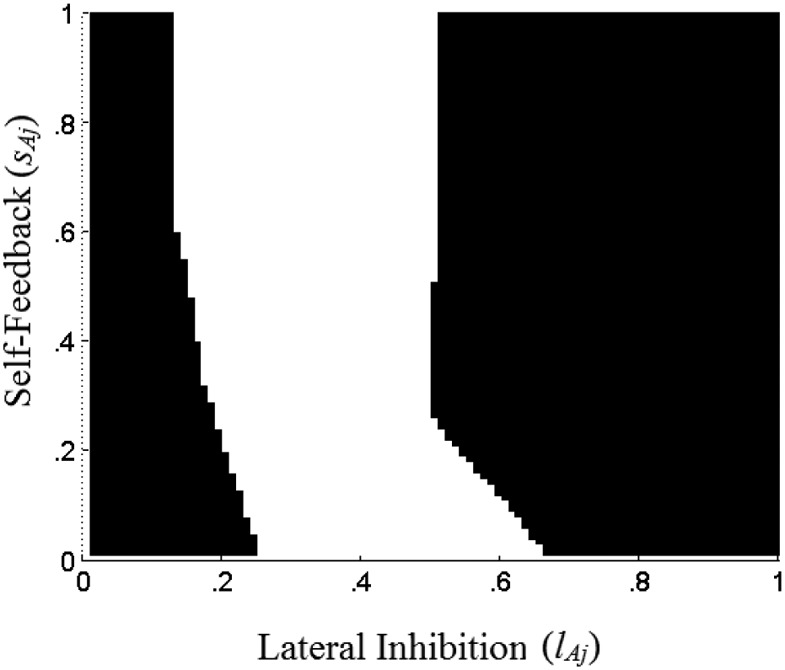
Parameter values identifying the worst alternative on an attribute *j* (α_*1j*_ > 0, α_*2j*_ > 0 and α_*3j*_ = 0) in the attribute representation sublayer *j*, as a function of *s*_*Aj*_ and *l*_*Aj*_. White values indicate parameter values that can make the correct identification. Here we have *x*_*1j*_ = 0.75, *x*_*2j*_ = 0.5, and *x*_*3j*_ = 0.25.

Figure 4 illustrates these insights, with *x*_*1j*_ = 0.75, *x*_*2j*_ = 0.5 and *x*_*3j*_ = 0.25. As in Figure 2, *s*_*Aj*_ and *l*_*Aj*_ are varied from 0 to 1. White areas on Figure 3 correspond to parameter combinations for which the network sublayer *j* is able to compute the worst alternative on attribute *j*. For these parameter combinations, we have equilibrium activations α_*3j*_ = 0, but α_*1j*_ > 0 and α_*2j*_ > 0. Parameter combinations shaded black, for low values of *l*_*Aj*_ give us α_*3j*_ > 0 (as well as α_*1j*_ > 0 and α_*2j*_ > 0), whereas parameter combinations shaded black, for high values of *l*_*Aj*_ give us α_*3j*_ = 0 but also α_*2j*_ = 0 (and α_*1j*_ > 0). A formal demonstration of how, given any vector of inputs ***y***_***j***_, we can find some values of *s*_*Aj*_ and *l*_*Aj*_ that implement these computations, is provided in the Appendix (see the online supplemental Appendix). [Fig-anchor fig4]

**Figure 5 fig5:**
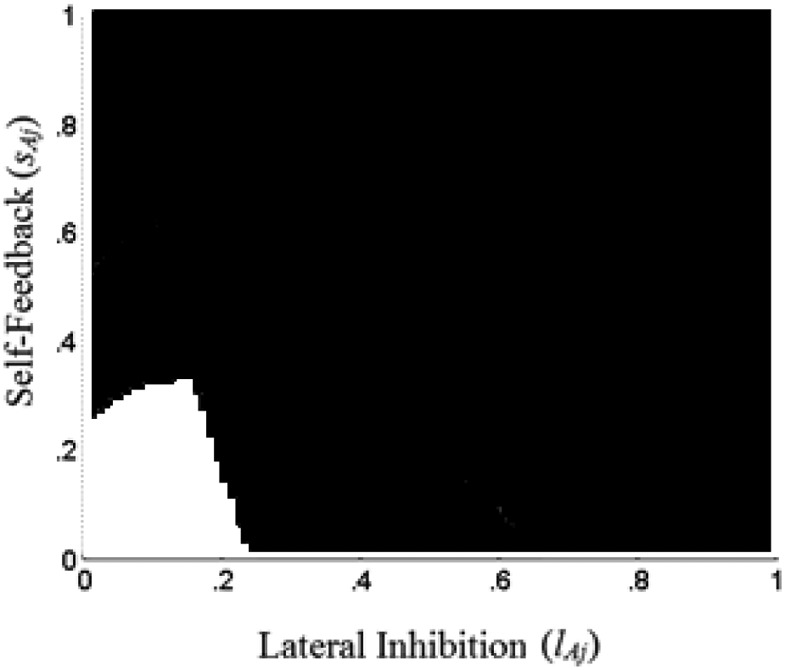
Parameter values that are able to normalize attributes ($\alpha_{ij}=\omega_{1}\;\cdot \;x_{ij}-\;\omega_{2}\;\cdot \;\sum_{k\neq i}x_{kj}$ where ω_1_ and ω_2_ are positive constants) in the attribute representation sublayer *j*, as a function of *s*_*Aj*_ and *l*_*Aj*_. White values indicate parameter values that can perform the correct normalization. Here we have *x*_*1j*_ = 0.75, *x*_*2j*_ = 0.5, and *x*_*3j*_ = 0.25.

There is also a third type of transformation that the proposed model can accomplish. This does not involve ordinal comparisons, but instead normalizes attributes based on the average attribute values in the choice set. Decision field theory (Busemeyer & Townsend, 1993; Roe et al., 2001), for example, assumes that decision makes do not accumulate absolute attribute values for each alternative, but rather attribute values relative to the average of all other alternatives on the attribute. This is typically accomplished by feed-forward inhibition. In the proposed model, however, it can also be accomplished by choosing appropriate values of *s*_*Aj*_ and *l*_*Aj*_, without requiring any feed-forward inhibition. Recall that low values of self-feedback increase the equilibrium activation of all nodes without saturating the activation of any node at 1. Likewise low values of inhibition reduce the equilibrium activation of all nodes without saturating the activation of any node at 0. The stable activation states that emerge from these settings are in fact relative, and the activation of any given attribute node can be written as a linear function that is increasing in its inputs but decreasing in the average inputs of all other nodes. More specifically, for these parameter combinations, we have equilibrium activations $\alpha_{ij}=\omega_{1}\;\cdot \;x_{ij}-\omega_{2}\;\cdot \;\sum_{k\neq i}x_{kj}$ where ω_1_ and ω_2_ are positive constants that depend on *s*_*Aj*_ and *l*_*Aj*_. This is nearly identical to the feed-forward inhibition based relative accumulation of decision field theory. Figure 5 illustrates these insights, with *x*_*1j*_ = 0.75, *x*_*2j*_ = 0.5 and *x*_*3j*_ = 0.25. As in Figure 2, *s*_*Aj*_ and *l*_*Aj*_ are varied from 0 to 1. White areas on Figure 5 correspond to parameter combinations for which the network sublayer is able to compute normalized attribute values for attribute *j*. A formal demonstration of how, given any vector of inputs ***y***_***j***_, we can find some values of *s*_*Aj*_ and *l*_*Aj*_ that implement these computations, is provided in the Appendix (see the online supplemental Appendix).[Fig-anchor fig5]

### Parameter Recovery

#### Parameter uniqueness

The above sections show that various parameter combinations on the attribute representation sublayers can perform ordinal and relativistic attribute transformations. This allows the proposed model to mimic a range of heuristic rules as well as mimic some existing accumulator models. One thing to note, however, is that the correspondence between parameter combinations and resulting behavior is not one-to-one. For a given choice set, there exist numerous parameters that are able to transform the attribute values in the choice set in a certain ordinal manner. For example, in Figure 3, we see that all *s*_*Aj*_ > 0.24 and *l*_*Aj*_ > 0.48 lead to stable activation states α_*1j*_ = 1, α_*2j*_ = 0 and α_*3j*_ = 0.

Whereas this property is fairly common in nonlinear systems, it can nonetheless be problematic from an empirical standpoint. It is difficult to use choice data to recover a decision maker’s underlying parameters, if numerous parameter values generate the same behavior. Fortunately, however, this problem is mitigated when the model is applied to multiple choices. Whereas different parameters can generate the same behavior when applied to one choice set, it is unlikely that they do so when applied to another. Thus data that contains choices made on a large number of choice sets can be used to accurately recover a decision maker’s true parameters.

In this section we demonstrate this insight in a parameter recovery study, which generates choice predictions from the model for various parameter values, and then attempts to recover these parameters using standard model fitting tools. We consider values of *s*_*Aj,*_ *l*_*Aj*_ and the threshold parameter *Q* in the interval [0.1, 0.9] in increments of 0.1, with *s*_*P*_ = 1 and *l*_*P*_ = 0. For simplicity we set self-feedback and inhibition to be identical for all sublayers so that *s*_*Aj*_ = *s*_*A*_ and *l*_*Aj*_ = *l*_*A*_. We consider three-alternative choice sets, with each alternative defined on two attributes. Our weights for these attribute are set to *w*_*1*_ = *w*_*2*_ = 0.01. Note these parameter values do not capture the LEX heuristic, thus we also consider one additional parameter combination^2^: *s*_*P*_ = *l*_*P*_ = 0, *s*_*Aj*_ = *l*_*Aj*_ = 1, and *Q* = *w*_*1*_ = 0.01. This set up gives us 9^3^ + 1 = 730 unique parameter combinations to examine in our parameter recoverability study.

The choice sets that we consider are generated randomly, with the attribute values for each alternative being drawn from a uniform distribution on the interval [0, 1]. We generate choice probabilities for our model for each of the 730 parameter combinations, on varying numbers of these randomly generated choice sets, and attempt to recover the true parameters based on the resulting data. Choice probabilities are generated by simulating the model on the choice sets 10,000 times. For our study we consider only other parameter combinations in our set of 730 considered parameter combinations, and attempt to find the parameter combinations that minimize the mean-squared-error on our data. Each parameter combination is simulated twice on each choice set: once to generate a choice probability for our data, and a second time to generate a choice probability for model fits on the data (note that we do not reuse the choice probabilities in our data when performing our model fits, so as to allow for noisy parameter recovery).

Using the above techniques we are able to specify a measure of the parameter uniqueness of our model for each combination of choices that it is applied to. We define parameter uniqueness as the proportion of the 730 parameter combinations that are recovered successfully and uniquely. We would obtain 100% parameter uniqueness if each of the 730 parameter combinations were the unique best-fit parameter combinations on the choice data that they generate. Parameter uniqueness would be less than 100% if two or more parameter combinations were both best-fit parameter combinations on the data (because they generate the same choice predictions), or if some parameter combination was not the best-fit combination on the data that it generated.

**Figure 6 fig6:**
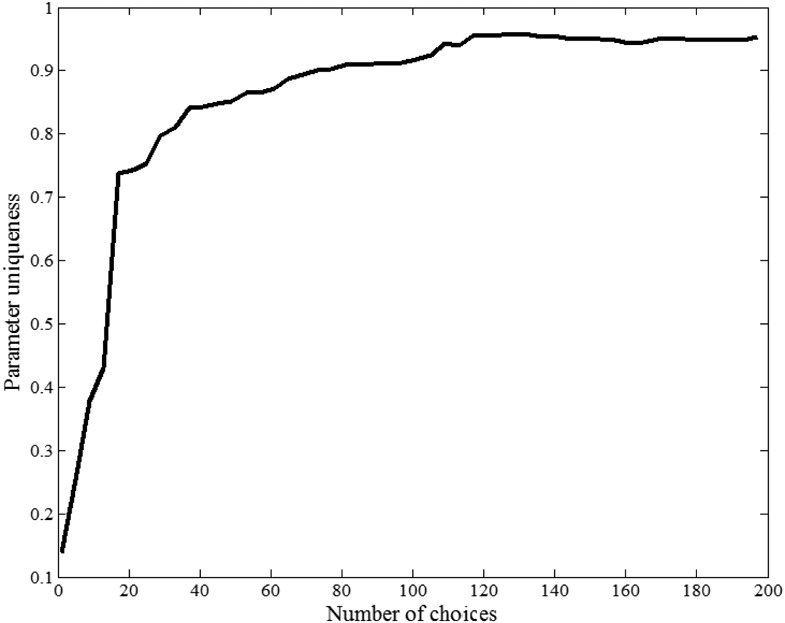
Parameter uniqueness as a function of the number of choices the model is applied to. Parameter uniqueness captures the proportion of considered parameter combinations that are the unique best-fit parameters to the data that they generate, in the parameter recovery study.

Figure 6 displays the parameter uniqueness of the proposed model as a function of the total number of randomly generated choice sets the model is applied to. Parameters are fairly nonunique and confusable when the model is applied to only a small number of choices. For example, only 101 out of the 730 parameter combinations give unique predictions when they are applied to two choices. In contrast 724 out of the 730 parameter combinations give unique predictions when they are applied to 200 choices. Thus the problem of parameter recovery is mitigated if the model is applied to a large dataset.[Fig-anchor fig6]

### Identifying Heuristics

Besides the parameter uniqueness issue discussed above, it is also not clear whether the proposed model can be used to identify the heuristic being used in any given setting. To what extent are the parameters that describe different heuristics confusable with each other? Can parameters that describe one heuristic also adequately describe other heuristics? This section addresses this issue with a simulation study. Particularly it applies the LEX, WP, MCD, EW, and WAD heuristics to 100 randomly generated three alternative choice sets. The available alternatives in these choice sets have three attributes, with attribute weights *w*_*1*_ = 0.03, *w*_*2*_ = 0.015 and *w*_*3*_ = 0.01. The attribute amounts in these alternatives are obtained using a uniform distribution with range [0, 1]. For each of the choice sets, this study generates choices made by idealized, deterministic versions of these heuristics (e.g., choices according to the LEX heuristic always choose alternatives with the highest values on Attribute 1). As it considers only deterministic choices, the CONF heuristic is excluded from the study.

This study then simulates the model on these 100 choice sets using five different combinations of parameter values. These five combinations correspond to the parameters that that allow the proposed model to mimic LEX, WP, MCD, EW, and WAD respectively (as shown in the above sections). The parameters used for LEX are *s*_*P*_ = *l*_*P*_ = 0, *s*_*Aj*_ = *l*_*Aj*_ = 1, *Q* = 0.03, *w*_*1*_ = 0.03, *w*_*2*_ = 0.015 and *w*_*3*_ = 0.01; the parameters used for WP are *s*_*P*_ = 1, *l*_*P*_ = 0, *s*_*Aj*_ = *l*_*Aj*_ = 1, *Q* = 0.99, *w*_*1*_ = 0.03, *w*_*2*_ = 0.015 and *w*_*3*_ = 0.01; the parameters used for MCD are *s*_*P*_ = 1, *l*_*P*_ = 0, *s*_*Aj*_ = *l*_*Aj*_ = 1, *Q* = 0.99, and *w*_*1*_ = *w*_*2*_ = *w*_*3*_ = 0.01; the parameters used for EW are *s*_*P*_ = 1, *l*_*P*_ = 0, *s*_*Aj*_ = *l*_*Aj*_ = 0, *Q* = 0.99, and *w*_*1*_ = *w*_*2*_ = *w*_*3*_ = 0.01; and the parameters used for WAD are *s*_*P*_ = 1, *l*_*P*_ = 0, *s*_*Aj*_ = *l*_*Aj*_ = 0, *Q* = 0.99, *w*_*1*_ = 0.03, *w*_*2*_ = 0.015 and *w*_*3*_ = 0.01. For each set of parameter values, the model is simulated 10,000 times to generate the choice probabilities predicted by these parameter combinations. These choice probabilities are compared with the choices generated by the actual idealized heuristics to test both the accuracy of the model parameters in mimicking the heuristics that they are assumed to mimic, and the extent to which these parameters can mimic other heuristics, and thus be confused with the parameters used to describe these others heuristics. Note that we have set the threshold *Q* to be considerably higher than the weights for all the heuristics (except for LEX) so that the model’s choices for these heuristics are made with almost no noise.

A combination of parameters is assumed to accurately describe a heuristic rule on a particular choice if the modal choice predicted by the set of parameters is the same as that generated by the idealized heuristic (the use of modal choices is necessary as the model is probabilistic but the heuristics being studied are deterministic). Parameter combinations fully describe a heuristic if their modal choice prediction is the same as the idealized heuristic’s prediction in all of the different choice sets being considered in this study.

The results of the simulation are summarized in Table 1, which presents the accuracy of each of the parameter combinations in capturing behavior generated by each of the idealized heuristics. In order to evaluate parameter accuracy for LEX, EW, and WAD we use all 100 of the randomly generated choice sets. However, to evaluate the parameter accuracy for MCD we use only 68 of the choice sets. This is because this heuristic does not make unique choice predictions on some choice sets where each alternative is the best on an equal number of attributes.[Table-anchor tbl1]

As expected, parameter combinations hypothesized to mimic an idealized heuristic can describe the behavior generated by the heuristic with 100% accuracy. In every case, they are unable to do so for other heuristics. However model predictions do sometimes overlap based on the similarities of the different heuristics. For example, EW and WAD are nearly identical heuristics except for their assumptions about weighting. The model is able to capture this relationship, by the relatively high accuracy of EW and WAD parameter combinations in describing idealized WAD and EW choices respectively. This is also the case for LEX, WP, and MCD, which all involve ordinal comparison on individual attributes.

## Discussion

A large number of heuristic rules, including the rules explored in this article, can be described in terms of the following three principles of bounded rationality: (1) The attributes of all alternatives are not evaluated. (2) The weighting of attributes is simplified. (3) Absolute attribute values are replaced with ordinal comparisons on the attributes. This article has shown that extending existing preference accumulation networks by assuming that leaky competitive accumulation is also at play in attribute representation, allows these networks to capture these three principles, and subsequently implement a number of heuristic rules based on these principles.

### A Neural Level of Analysis

An important research tradition in psychology involves understanding cognition and behavior using parallel distributed processes (Rogers & McClelland, 2014; Rumelhart, McClelland, & the PDP Research Group 1986). Models within this framework formalize cognitive processes using ensembles of neuron-like units, in an attempt to understand how a range of psychological tasks could be performed by the human brain. The model presented in this article is a product of this research tradition. By studying the emergence of sophisticated heuristic rules using a connectionist model, it shows how these important heuristic rules can be captured using the key assumptions of the connectionist framework; assumptions that stem from the neurophysiological properties of the brain, and assumptions that have already been used to successfully model a diverse array of cognitive and behavioral findings.

The model in this article most directly relates to the leaky competitive accumulation model proposed by Usher and McClelland (2001), and further applied to preferential choice by Usher and McClelland (2004). As in these models, this article considers neural networks with lateral inhibition and self-feedback. Both of these types of recurrent connections are fundamental properties of neurophysiological processing, and both inhibition and feedback are frequently assumed be active in psychological neural networks (e.g., Amari, 1977; Elman, 1990; McClelland & Rumelhart, 1981; see also Usher & McClelland, 2001 for a detailed discussion). Indeed, in their 2001 article, Usher and McClelland suggested that studying how leakage and competition play out in complex multilayer decision models is the reasonable next step in their research agenda (see p. 554). The heuristic model proposed here directly addresses this issue: it shows that multilayer networks composed of leaky competitive accumulators are able to generate the types of sophisticated computations associated with symbolic heurist ic rules (see also Hunt, Dolan, & Behrens, 2014 for a related approach).

Extending the assumption of leakage and competition to multilayer accumulation networks primarily involves formally modeling attribute-level connectivity, which is an assumption that is largely absent in existing accumulation networks. Interattribute connectivity is necessary to understand how the attribute values of one alternative influence how those of another are processed. In this article, it is the key mechanism that allows the model to generate ordinal transformations of attribute values, which are necessary to implement the various heuristic rules within the proposed framework.

Within-attribute connectivity also allows the model to normalize attributes based on the average value of other attributes. As discussed in an earlier section in this article, attribute normalization is a key assumption in many accumulator networks, including decision field theory (Roe et al., 2001). Decision field theory and related approaches normalize attributes using feed-forward connectivity between the attribute layer and a valence processing layer. For an *M* attribute and *N* alternative choice space, this requires a total of *M* · *N*^2^ feed-forward connections, as well as a separate layer to process these connections. This article’s assumption of within-attribute leaky competitive connectivity is comparatively more parsimonious. It requires only *M* · *N* feed-forward connections and does not need an additional layer. This assumption is also biologically plausible, as considerable evidence suggests that inhibitory connections are primarily of the lateral type (again, see Usher & McClelland, 2001 for an overview).

### Other Heuristic Networks

This article is not the first to implement heuristic choice rules in a neural network. Usher and Zakay (1993) present an attractor neural network with dynamic thresholds, which can approximate some of the heuristics discussed above. This article is, however, the first to implement heuristic choice rules in leaky competitive accumulation networks, which are, as discussed above, are often considered to provide realistic descriptions of the neurocognitive underpinnings of preferential choice (Roe et al., 2001; Usher & McClelland, 2004). This model is additionally able to implement heuristic rules that rely on ordinal processing. That said, the approaches discussed in this article and in Usher and Zakay (1993) are complementary. Allowing for dynamic thresholds in attribute activation can, for example, provide a neurocomputational justification for the sequential sampling assumption made in this article. Besides introducing additional biological realism to the proposed model, these thresholds can also be used to predict when the sequential sampling assumption will be violated, thereby expanding the model’s descriptive scope.

This article is also related to a connectionist framework proposed by Glöckner and Betsch (2008a). Glöckner and Betsch suggest that preferential choice involves both an automatic system, which guides the integration of information, and a deliberative system, which guides information search. In their framework, both the automatic and deliberative systems are modeled using neural networks, though the deliberative system can be seen as processing heuristic rules for information search. The model proposed in this article can benefit from many of the insights presented in Glöckner and Betsch (2008a). This model does not, for example, specify the determinants of attribute sampling. It is highly likely that a secondary network, similar to the deliberative system proposed by Glöckner and Betsch, is involved at this stage. Likewise the proposed model does not feature feedback from preferences to attributes, a feature that a natural part of many neural network modeling approaches, including the one proposed by Glöckner and Betsch (2008a).

Note that there are a number of behaviors that are predicted by heuristic network approaches such as Glöckner and Betsch (2008a), which contradict the assumptions of symbolic heuristic approaches. These include findings regarding information intrusion (Söllner, Bröder, Glöckner, & Betsch, 2014), eye-tracking behavior (Glöckner & Herbold, 2011), and the integration of multiple attributes (Glöckner & Betsch, 2008b). Generally these findings indicate that decision makers sample more than a single attribute when making their choices. The proposed model can capture these findings if the threshold parameter *Q* is set to be sufficiently high.

### Decision by Sampling

The proposed model is able to implement heuristic rules in part by exploiting the ability of leaky competitive accumulator layers to make ordinal comparisons between attribute values. This property of the model can also allow it to implement other choice rules that are not traditionally considered to be heuristics. One such rule involves rank based comparison, as specified by the decision by sampling model (Stewart, Chater, & Brown, 2006; Stewart & Simpson, 2008). According to the decision by sampling model, decision makers evaluate an alternative using its rank within a sample of attribute values. Particularly, at each time step decision makers choose an attribute and draw a sample of a value on this attribute from memory. The value of the considered alternative on this attribute is then compared against the memory sample. If it is higher than the memory sample, the preference for the alternative increases by one unit; if not, the preference remains the same. Ultimately, decision makers aggregate the total number of attribute comparisons on which the considered alternative is better than the alternatives stored in memory, a number that is closely associated with the relative rank of the considered alternative in the memory sample. One of the most powerful insights of the decision by sampling model is that this relative rank can be used to explain the cognitive basis of nonlinear value scales, as well as to predict the way in which these scales differ across attribute and choice domains.

The decision by sampling model can be implemented in the proposed neural network with additional assumptions about how nonavailable comparison alternatives are selected. We can, for example, assume that decision makers randomly send inputs into the nodes of nonavailable alternatives in the attribute representation layer, in each time period. With appropriate self-feedback and inhibition parameters, decision makers would then be able to make ordinal judgments involving the considered alternative and its nonavailable comparison sample. These judgments would be aggregated in the preference accumulation layer in much the same way as the MCD and WP heuristics. Whereas such a model differs in important ways from the network discussed in this article, it nonetheless provides valuable insights about the neurocognitive basis of decision by sampling, and the ways decision by sampling relates to both existing heuristic choice rules and existing models of preference accumulation.

### Heuristic Selection

Although the proposed network is able to accurately generate a number of existing heuristic rules, it relies on fundamentally different properties than the algorithmic approaches previously used to model these rules. One implication of this is that measures of effort assumed by these existing approaches (e.g., Johnson & Payne, 1985; Payne et al., 1993) do not translate easily into the proposed framework. As a result, this network may not be able to generate effort-accuracy relations, and subsequent results regarding heuristic learning and selection, obtained in prior work.

That said, the proposed network does provide novel insights regarding heuristic learning and selection. The heuristics emerging from the dynamics of the network depend, for example, on both the parameters of the network and the attribute values of the alternatives being considered. Parameters that implement a particular heuristic, for a given choice set, may not implement this heuristic for a different choice set. Thus the properties of the proposed network are not only parameter dependent, but also alternative dependent. This dependence allows the network to learn heuristics based on the reward structures of the domains (that is, the choice sets) it is applied to (see Payne et al., 1988). A learning rule which changes connection weights as a function of the experienced value of the chosen item, the bias and variance involved in predicting this experienced value, and also possibly the time taken to make the decision, will often be able to determine the heuristic that is best-adapted for use in a given domain, thereby generating behavior that is not only boundedly rational, but ecologically rational as well (Goldstein & Gigerenzer, 2002).

This property of the model can also allow it to learn the settings where it is best not to use any heuristic at all. Both heuristics and standard accumulators networks (such as decision field theory) are in the mind’s toolbox, and will perform differently under different conditions. As shown above, these preference accumulation networks are a special case of the proposed model, implying that the model could learn to generate behavior resembling these accumulator networks, when this behavior is adaptive. Further work should examine how this type of learning can be accomplished, and whether it differs in important ways from existing theories of heuristic selection (e.g., Marewski & Schooler, 2011; Payne et al., 1993).

### Stochastic Choice and Decision Time

Another important difference between the proposed model and current approaches to studying heuristic choice involves probabilistic choice and decision time predictions. Many heuristic rules are deterministic in nature, though choice, of course, is probabilistic. Additionally, the probabilistic nature of choice extends beyond variability in the final chosen option; it applies also to the time that is taken to make the decision. Modifying the heuristic framework to allow for stochastic and dynamic choice, in a cognitively plausible manner, is an important challenge for decision researchers (see, e.g., Bröder & Schiffer, 2003).

The proposed model addresses this challenge. Accumulator networks are stochastic and dynamic and are subsequently able to make powerful predictions regarding choice probabilities and their associations with decision time (see, e.g., Busemeyer & Townsend, 1993). By embedding heuristic rules within an accumulator framework, the proposed model provides a useful new extension of the heuristic framework. This extension involves more than just adding random additive noise or tremble noise to the output of a heuristic. Noise is a fundamental feature of preference accumulation, and interacts in sophisticated ways with both the choice options in the available decision set and the time that the model takes to decide between these options. The proposed instantiation of LEX, for example, links decision time and error to the height of the threshold. Thresholds that are smaller than the weight placed on the most important attribute will, at times, lead to the selection of alternatives that are the best on the second or third most important attribute. Decisions with these reduced thresholds will also, on average, be quicker than decisions when the threshold is set at a higher level. Likewise, decision time and error are both a function of the threshold for heuristics like MCD. This relationship is systematic: With small enough thresholds, errors in MCD generate behavior that resembles k-CONF. Finally, as with other preference accumulation models (Roe et al., 2001; Usher & McClelland, 2004), choice probability is sensitive to the similarity of the options within the choice set. This implies that the probabilistic behavior of heuristics like EW and WAD should depend strongly on the overlap of the attributes in the alternatives that the decision maker is presented with.

The dynamics and the stochasticity of the proposed model allow it to generate new testable predictions that cannot be accommodated within the classical heuristic framework. These predictions are necessary to differentiate the proposed model from existing heuristics. They are also valuable for fitting heuristic models to choice data, and for comparing heuristic and nonheuristic approaches using more than just choice data. This type of examination should be the focus of future work.

### Novel Rules

The rules discussed in this article are only a small portion of the entire set of rules that can be generated by the proposed framework. Novel decision rules can easily be obtained by combining the various features of these heuristics. Allowing for decision criteria to differ across attributes can also create alternate, potentially more complex rules. Whereas many of these novel rules may not fit the intuitive algorithmic structures of LEX, CONF, WP, MCD, EW, or WAD, they may prove to be equally (if not more) suitable for describing choice behavior. These rules may also prove to be theoretically desirable, instantiating properties not easily captured by current heuristic structures.

Consider for example a decision rule called ignore the worst (ITW). Such a rule considers the values of the available alternatives on the various attributes, but then aggregates only the values of the alternatives that are not the worst on the attribute dimension being examined. Another possible rule is *k* majority of confirming dimensions (k-MCD) which tallies up ones and zeros based on whether an alternative is one of the *k*-best on an attribute. Such rules may be used by decision makers, but current heuristic approaches offer no way of predicting or fitting behavior that is generated by these rules. Both these types of rules can be generated by the proposed model, and examining whether decision makers can use these rules provides another way to test the proposed model against existing heuristic approaches.

### Feasible Rules

Is the proposed network flexible enough that it can implement any heuristic rule? No is it not. For example, the network is unable to implement a dominance based heuristic, which only selects options if they dominate others (e.g., Hogarth & Karelaia, 2005b). Such a heuristic requires decision makers to keep track of ordinal attribute relationships between each pair of options, which is something that an accumulator model cannot easily do. Likewise the current model cannot implement heuristics that involve elimination, such as fast and frugal trees or the elimination by aspects heuristic (Dhami, 2003; Luan et al., 2011; Tversky, 1972).

In general there are countless other rules, such as those involving different decision criteria for different alternatives, comparisons across different attributes, or comparisons between multiple pairs of alternatives, that also cannot be implemented in the proposed network. Whereas some may consider this to be a limitation of the model, we believe that it is highly desirable. The space of possible heuristic choice rules is incredibly vast and the unconstrained application of these rules to model behavior can lead to problems of overfitting and generalizability. By making predictions about the types of choice rules that are likely to be observed, and more important unlikely to be observed, the proposed model adds valuable theoretical precision to the study of heuristic choice.

For example some prior work assumes that elimination may be used in early stages in tasks involving large choice sets, to reduce the number of alternatives being considered (see, e.g., Roe et al., 2001 for a discussion). As elimination requires a lower rejection threshold, which is not a feature of the current model, the model does not generate this behavior. In the current form the model predicts that all alternatives should be considered throughout the choice process. All of these alternatives should affect choice because of the recurrent connectivity on the attribute representation sublayers, though relatively undesirable alternatives should still be less likely to be chosen. Predictions such as these provide another way of comparing the proposed model with exiting heuristic approaches. Once again, this should be the focus of future empirical work.

### Elementary Information Processes

Choice can be decomposed into a set of basic cognitive components, or elementary information processes. Cognitive research on decision making has for a long time been concerned with the identification of these processes. Early work in this area (see, e.g., Johnson & Payne, 1985) used insights from Newell and Simon’s (1972) approach to studying problem solving, and formulated these elementary processes using symbolic rules implemented in production systems. This article finds that these rules can be further decomposed into more basic neurocomputational components. This suggests that the elementary information processes underlying decision making are not production rules applied to abstract symbols; rather they are accumulator networks with leakage and competition.

Why do we care about the uncovering the elementary information processes underlying choice? First, such an exercise can provide important organizing principles for heuristics research. In this article we have shown how the various properties of our preference accumulator network correspond to three basic principles of heuristic choice. These properties can be used to categorize different heuristics (based on differences in the parameters involved in their implementation), which in turn can help uncover the various statistical and behavioral regularities involved in the use of these heuristics.

Understanding elementary information processes can also provide rich insights for existing preference accumulation networks (Bhatia, 2013; Roe et al., 2001; Usher & McClelland, 2004). These models are theoretically desirable. They are both neurally feasible, and are able make optimal decisions. In this article we provide new results regarding the algorithmic power of these networks. Preference accumulation networks are not only valuable for their neural and statistical properties; they can also generate the range of complex, sophisticated, structured strategies known to be at play in preferential choice.

## Conclusion

Heuristic choice rules specify short cuts for making decisions. Thus far, research on heuristic choice has focused largely on describing the rules used by decision makers (Gigerenzer et al., 1999; Payne et al., 1993; Simon, 1956; Tversky & Kahneman, 1974), or on outlining the statistical properties of these rules (Davis-Stober, Dana, & Budescu, 2010; Hogarth & Karelaia, 2007; Pachur, 2010). Here we show how a number of different heuristic rules can be implemented in dynamic connectionist networks, particularly networks that accumulate attribute values into preferences with leakage and competition (Bhatia, 2013; Roe et al., 2001; Usher & McClelland, 2004). Preference accumulation networks are biologically realistic and provide important insights regarding choice behavior in a number of domains. By extending these networks to incorporate heuristic rules, we allow for the integration of two of the most popular theoretical approaches to studying multi-attribute choice, an integration that allows for insights from each of these approaches to be transferred to the other, and demonstrates the possibility of a unitary, cohesive cognitive theory of preferential decision making.

## Supplementary Material

10.1037/dec0000038.supp

## Figures and Tables

**Table 1 tbl1:** Accuracy of Various Parameter Combinations Used to Mimic the Heuristic Rules in Describing Choices Generated by Idealized Heuristic Rules

Implemented heuristic	Idealized heuristic				
	LEX	WP	MCD	EW	WAD
LEX	1.00	0.85	0.78	0.59	0.68
WP	0.85	1.00	0.93	0.71	0.72
MCD	0.75	0.89	1.00	0.73	0.71
EW	0.59	0.71	0.84	1.00	0.87
WAD	0.68	0.72	0.82	0.87	1.00
*Note.* The accuracy of different parameter combinations are displayed across rows. Accuracy is defined by the proportion of unique choices correctly captured using modal model choice predictions. MCD = majority of confirming dimensions; EW = equal weights heuristic; WAD = weighted additive rule.					

## Footnotes

1 Note that stability is guaranteed through the Cohen–Grossberg theorem (Cohen & Grossberg, 1983). Although *f* is not differentiable at 0 and 1, a requirement for the theorem, we can replace *f* with an appropriate differentiable approximation, while maintaining all the properties of the accumulation network, discussed in this article.

2 The model will not cross the threshold if *s*_*p*_ = 0 with *Q* > *w*_*1*_ or if *s*_*p*_ = 0 for small enough *s*_*A*_. Thus, to have well-defined predictions for parameters capable of mimicking LEX heuristic, we need to specify these parameters separately.
