# Supplementary material for: Choice Rules and Accumulator Networks
Source: Decision (Wash D C ). 2015 Jul 27;4(3):146–70. doi: 10.1037/dec0000038 (PMC5484390; doi:10.1037/dec0000038)
Supplement: Supplementary file 1 [file dec-DEC-2014-1053-Appendix.pdf]

Appendix

This appendix describes values of s and l that are able to make best/worst and normalized comparisons in the attribute representation sublayers. The parameter values listed here are not exhaustive (this discussion serves largely as an existence proof). The time scale used in this discussion will be the time scale of the attribute representation layer, and for all t the external inputs to the nodes in the sublayer will be $\mathbf{y} = (x_1, x_2, \ldots, x_n, 0, 0, \ldots, 0)$, with $1 > x_1 > x_2 > \ldots > x_n > 0$. The activation of a node corresponding to alternative i at time t will be written as $A_i(t)$ and, with the piecewise linear function f, defined in the text, its dynamics will be determined by:

$$A_i(t) = f[s \cdot A_i(t-1) - l \cdot \sum_{k \neq i} A_k(t-1) + x_i] \quad (1)$$

There are three different types of attribute sublayer computations discussed in this paper. One requires the identification of the worst alternative on an attribute with stable activation state α such that $\alpha_i > 0$ for $i < n$ and $\alpha_i = 0$ for $i = n$. These computations can be understood by considering the stable activation state of the system in the absence of upper and lower bounds imposed by f, and in the absence of nodes $i > n$, whose inputs are 0. Without the bounds the system is linear. At time t, the activations of the nodes in this system can be represented by the n dimensional vector $\mathbf{A}(t)$, which is transformed across time by the $n \times n$ state transition matrix W, with $W_{ij} = s$ for $i = j$ and $W_{ij} = -l$ for $i \neq j$. W is a symmetric toeplitz matrix, and its eigenvalues are $\lambda_1 = s - (n-1) \cdot l$ for the first eigenvector and $\lambda_2 = s + l$ for all other eigenvectors (Makhoul, 1981). Requirements for stability are $\lambda_1, \lambda_2 \in (-1, 1)$. Let us assume that s and l are such that these requirements are satisfied. We can thus write the asymptotic activation of node i as:

$$\alpha_i = \frac{x_i}{1-\lambda_2} - \left[\frac{1}{1-\lambda_2} - \frac{1}{1-\lambda_1}\right] \cdot \frac{1}{n} \cdot \sum_{k=1}^{n} x_k \quad (2)$$

Now for simplicity let us set $s = 0$, and find a value of l such that $\alpha_n = 0$. Imposing these restrictions on equation 2 and simplifying gives us:

$$l = \frac{C}{1 - (n-1) \cdot C} \quad (3)$$

Here $C = \frac{x_n}{\sum_{k=1}^{n} x_k}$. As $C < \frac{1}{n}$, equation 3 guarantees some $l > 0$. Also note that the stability requirements imply that $|s - (n-1) \cdot l| < 1$, which means that $l < \frac{1}{n-1}$. Placing equation 3 into this inequality gives us $n < \frac{2}{C} + 1$. This is guaranteed for any n, as $C < \frac{1}{n}$.

Now we have found a value of l such that for $s = 0$, the system without upper or lower

bounds, and without nodes $i > n$, stabilizes with activation $\alpha_n = 0$. As $s = 0$, the stable activations of all other nodes $i < n$, is $0 = \alpha_n < \alpha_i < x_i < 1$. Thus for these nodes we do not need to worry about the bounds imposed by f. With the lower bound on f, we also have $\alpha_i = 0$ for $i > n$. This means that the activations of these nodes remain at 0, and these nodes can be ignored from the analysis. Thus the value of l (with $s = 0$) that gives us the required computation without bounds and without nodes $i > n$ also gives us this computation with these bounds and with these nodes.

The second type of computation considered in this paper requires the identification of the best alternative on an attribute, with stable activation state α such that $\alpha_i = 1$ for $i = 1$ and $\alpha_i = 0$ for $i > 1$. For this analysis, let us explore the restrictions we can place on $A_i(t)$ over time. At $t = 1$ we have $A_i(1) = x_i$. At $t = 2$ we have $A_i(2) = f[s \cdot x_i - l \cdot \sum\limits_{k \neq i} x_k + x_i]$. To ensure that we are able to perform best comparisons, we can impose the following restriction: $A_1(2) > 0$ and $A_i(2) = 0$ for $i > 1$. The latter is guaranteed if $A_2(2) = 0$. Thus our restrictions can be written as:

$$s \cdot x_1 - l \cdot \sum_{k \neq 1} x_k + x_1 > 0 \tag{4}$$

$$s \cdot x_2 - l \cdot \sum_{k \neq 2} x_k + x_2 \leq 0 \tag{5}$$

The constraints imposed by equations 4 and 5 can be simplified into equations 6 and 7 respectively

$$s > \frac{l}{A} - 1 \tag{6}$$

$$s \leq \frac{l}{B} - 1 \tag{7}$$

Here we have $A = \frac{x_1}{\sum\limits_{k \neq 1} x_k}$ and $B = \frac{x_2}{\sum\limits_{k \neq 2} x_k}$. Note that we have $A > B > 0$ and also that $B < 1$. This implies that values of s and l satisfying the above constraints can be seen on the s/l plane, as lying between two lines with positive slope, both originating at $s = -1$. For a large enough s, some value of l that satisfies these constraints necessarily exists, and similarly for a large enough l some value of s that satisfies these constraints also necessarily exists.

Now, equations 6 and 7 are not sufficient to ensure that $A_i(3)$ are suppressed to 0 for all $i > 1$. This restriction is however guaranteed if $A_2(3) = 0$, which imposes a further constraint, written in equation 8.

$$x_2 < l \cdot A_1(2) = l \cdot f[s \cdot x_1 - l \cdot \sum_{k \neq 1} x_k + x_1] \tag{8}$$

Now let us assume that $s \cdot x_1 - l \cdot \sum\limits_{k \neq 1} x_k + x_1 \leq 1$ implying that $A_1(2) = s \cdot x_1 - l \cdot \sum\limits_{k \neq 1} x_k + x_1$. The constraint written in equation 8 can now be rewritten as:

$$x_2 < l \cdot [s \cdot x_1 - l \cdot \sum_{k \neq 1} x_k + x_1] \tag{9}$$

This simplifies into

$$s > \frac{C}{l} + \frac{l}{A} - 1 \tag{10}$$

Here $C = \frac{x_2}{x_1}$ and A is as defined above. Now as l becomes large, $\frac{C}{l}$ approaches 0, and the constraint in equation 10 converges to the constraint in equation 6, which is necessarily satisfied for a correspondingly large enough value of s. If we have $s \cdot x_1 - l \cdot \sum\limits_{i \neq 1} x_i + x_1 > 1$ then the constraint in equation 8 is merely $x_2 < l$ which is also satisfied for a large l (and a correspondingly large s).

Now we have thus far shown that some large enough values of l and s exist so that we have $A_1(2) > 0$ and $A_i(2) = 0$ for all $i > 1$, and also that $A_i(3) = 0$ for all $i > 1$. Now if we select any $s \geq 1$ then we have $A_1(3) \geq A_1(2) > 0$, which in turn is sufficient to ensure that $A_i(t) = 0$ for all $i > 1$ and all $t > 3$. $s \geq 1$ also ensures that for large enough t we obtain $A_1(t) = 1$. Note that for the required $s \geq 1$, a large enough value of l can be found, so that the constraints necessary to ensure $A_1(2) > 0$, $A_i(2) = 0$ and $A_i(3) = 0$ for all $i > 1$, are satisfied. For these values of s and l our network can perform the required best computations.

The final type of computation considered in this paper requires normalizing attribute values, so that $\alpha_i = \omega_1 \cdot x_i - \omega_2 \cdot \sum_{k \neq i} x_k$ where ω_1 an ω_2 are positive constants. Some simple algebra shows that rewriting equation 2 can give us this form, or more specifically, can give us $\alpha_i = [\omega_3 - \omega_2] \cdot x_i - \omega_2 \cdot \sum_{k \neq i} x_k$, where $\omega_3 = \frac{1}{1-\lambda_2} > \omega_2 = \left[\frac{1}{1-\lambda_2} - \frac{1}{1-\lambda_1}\right] \cdot \frac{1}{n} \geq 0$ for $\lambda_1, \lambda_2 \in (-1, 1)$. If $s = l = 0$ then we have $\omega_3 = 1$ and $\omega_2 = 0$. If we increase s and l marginally, then we get our required form, without $\alpha_1 \geq 1$ or $\alpha_n \leq 0$. This gives us the required stable activation state in the presence of bounds on f.
